# Microglia: A Double-Edged Sword in Intracerebral Hemorrhage From Basic Mechanisms to Clinical Research

**DOI:** 10.3389/fimmu.2021.675660

**Published:** 2021-05-06

**Authors:** Jiachen Liu, Lirong Liu, Xiaoyu Wang, Rundong Jiang, Qinqin Bai, Gaiqing Wang

**Affiliations:** ^1^ Xiangya Medical College of Central South University, Changsha, China; ^2^ Department of Neurology, Shanxi Medical University, Taiyuan, China; ^3^ Department of Neurology, Sanya Central Hospital (Hainan Third People’s Hospital), Sanya, China

**Keywords:** intracerebral hemorrhage, microglia, phenotype switch, neuroimmunology, neuroinflammation

## Abstract

Microglia are the resident immune cells of the central nervous system (CNS). It is well established that microglia are activated and polarized to acquire different inflammatory phenotypes, either pro-inflammatory or anti-inflammatory phenotypes, which act as a critical component in the neuroinflammation following intracerebral hemorrhage (ICH). Microglia produce pro-inflammatory mediators at the early stages after ICH onset, anti-inflammatory microglia with neuroprotective effects appear to be suppressed. Previous research found that driving microglia towards an anti-inflammatory phenotype could restrict inflammation and engulf cellular debris. The principal objective of this review is to analyze the phenotypes and dynamic profiles of microglia as well as their shift in functional response following ICH. The results may further the understanding of the body’s self-regulatory functions involving microglia following ICH. On this basis, suggestions for future clinical development and research are provided.

## Introduction

**Figure 1:**
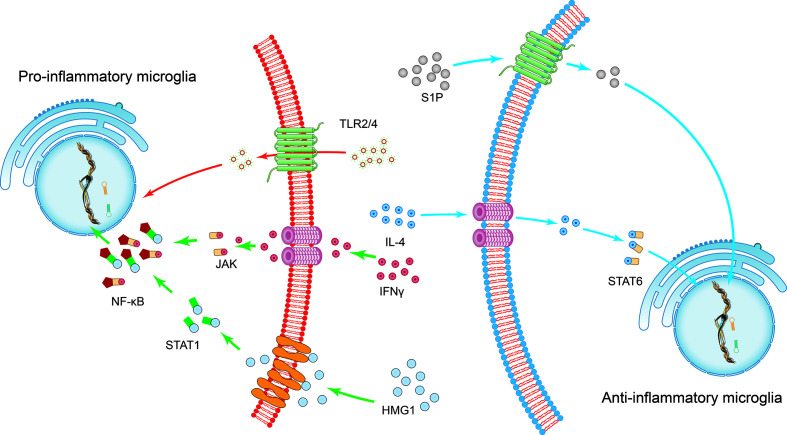
The induction of microglial polarization by several transcription factors. Activation of high mobility histone 1 (HMG1) and Toll-like receptor (TLR) 2 or TLR4 promotes the pro-inflammatory phenotypic polarization of microglia. Interferon-gamma (IFN-γ) promotes the pro-inflammatory phenotypic polarization of microglia through the signaling sensors signal transducer and activator of transcription (STAT) 1 and Janus kinase (JAK). This series of processes involves the nuclear factor kappa B (NF-κB) signaling pathway. On the other hand, STAT6 accumulates under the action of IL-4 and is responsible for the transcription of M2 phenotype-related genes. The sphingosine-1-phosphate (S1P) receptor signaling pathway downregulates the expression of pro-inflammatory cytokines and enhances anti-inflammatory responses following intracerebral hemorrhage.

Microglia constitute 5% to 10% of adult brain cells and form the largest group of immune cells in the CNS (1). The primary source of microglia is yolk sac erythromyeloid precursors (EMPs) that migrate into the brain rudiment during embryo development (2). Under physiological conditions, microglia self-renew for the entire lifespan of the organism and interact with numerous other cells in the brain, such as astrocytes, neurons, and oligodendrocytes (3). Mounting evidence suggests that microglia, as brain resident immune cells, play an essential role in maintaining normal brain function. When pathologic changes disrupt homeostasis in the brain, microglia are activated to exert regulatory effects (4). Microglia are highly diverse, and their phenotype depends on the context and type of stressor or pathology (5). Specifically, during the different periods after intracerebral hemorrhage (ICH), microglia may polarize to produce pro-inflammatory mediators or acquire a more anti-inflammatory phenotype, which has a decisive influence on ICH progression (**Figure 1**) (6).

Pathological analysis of microglial activity during ICH has revealed that microglia induce potent immune responses after extravasation of blood into the brain (7). The strong inflammatory response in microglia is caused by the rapid accumulation of blood-derived products (e.g., hemoglobin, heme, and iron) after ICH (8), which can directly damage the brain parenchyma, and a sustained microglia-mediated inflammatory response results in neurologic deterioration (9). Interestingly, Chang et al. (10) have experimentally proven that as early as 1 to 1.5 h after ICH, microglia respond to hemorrhagic injury and exhibit a protective alternative activation phenotype.

**Figure 2:**
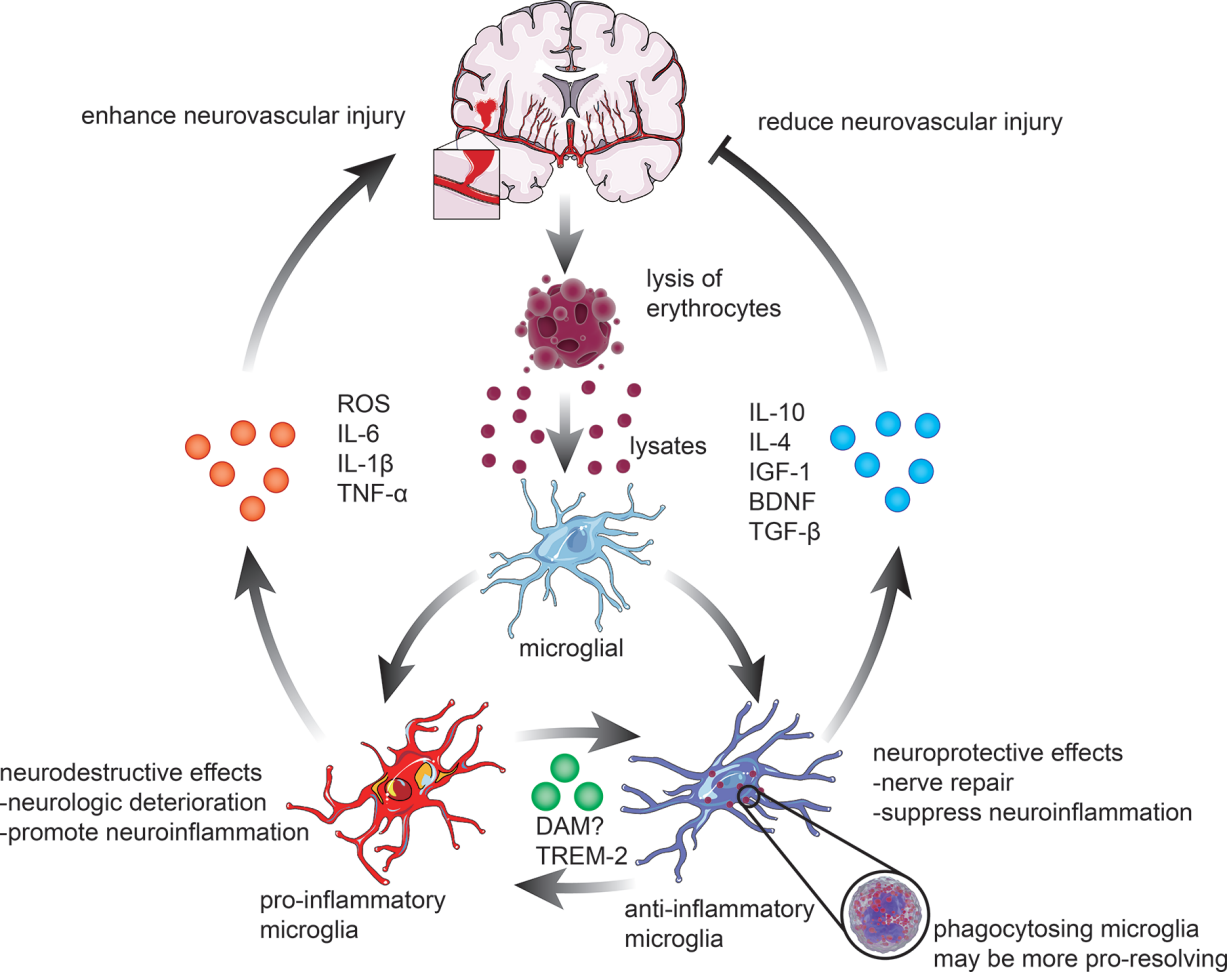
Microglial activation and polarization following ICH. The polarization of microglia in response to stimulation with erythrocyte lysates after ICH can be broadly classified into two categories. 1) pro-inflammatory microglia elevate the levels of ROS, interleukin-6 (IL-6), interleukin-1β (IL-1β), and tumor necrosis factor-α (TNF-α), hence enhancing the pro-inflammatory and destructive effects of ICH on the brain. 2) anti-inflammatory microglia, on the contrary, mainly exert neuroprotective effects, including actions related to nerve repair and anti-inflammatory effects, through phagocytosis of lysates, which are always associated with higher expression of IL-10, IL-4, insulin-like growth factor-1 (IGF-1), brain-derived neurotrophic factor (BDNF), and transforming growth factor-β (TGF-β). In recent years, an increasing number of studies have revealed that microglia can also be polarized into neuroprotective and neurodestructive phenotypes, such as disease-associated microglia (DAM) and triggering receptor expressed on myeloid cells 2 (TREM2) phenotypes, which remain to be further investigated.

The role of microglia following ICH is complex, and the entirely different action of different phenotypes of microglia plays an essential role in the development of cerebral inflammatory injury and recovery of the brain after ICH (**Figure 2**). Besides, the neuroprotective effect of microglia may serve as promising targets for ICH treatment. For example, the primary role of activated microglia is to phagocytose the hematoma, thereby reducing ICH-induced brain swelling and neuronal loss and improving neurological deficits (11). Microglial depletion can lead to more severe brain swelling, neuronal loss, and functional defects following ICH (12).

The dual role of reactive microglia in inflammation processes has a biphasic influence on the brain, which acts as a double‐edged offensive and defensive sword in brain injury. This paper reviews recent empirical studies on microglial activity following ICH to identify the most critical factors that influence this process, offering a fresh perspective for developing novel therapeutic strategies.

## The Dual Role Of Microglia After Ich

### The Effects of Microglia on the Blood-Brain Barrier Following ICH

Destruction of the blood-brain barrier (BBB) and consequent brain edema is the most common secondary causes of life-threatening events after ICH (13). One recent study by Chen et al. showed that microglia-derived TNF-α mediates endothelial necroptosis contributing to blood-brain-barrier disruption (14). Besides, activated microglia cause an imbalance between endogenous vasodilators and vasoconstrictors, further leading to edema formation after ICH (15).

Anti-inflammatory actions of microglia may mediate BBB protection and neural repair by producing anti-inflammatory cytokines, extracellular matrix proteins, glucocorticoids, and other substances (16). As signaling *via* IL-4 and IL-10 can induce an anti-inflammatory phenotype of microglia, it can be targeted for ICH treatment through modulating BBB physiology (17).

### The Functions of Microglia in Secondary Brain Damage Following ICH

**Figure 3:**
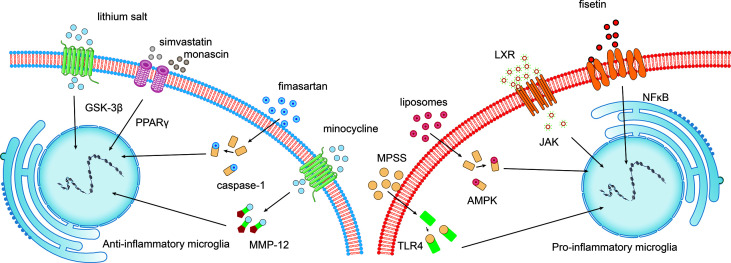
Potential intervention strategies targeting the microglial phenotypic shift following ICH. Interventions that induce the polarization of pro-inflammatory microglia towards the anti-inflammatory phenotype exert beneficial effects following ICH. For example, fisetin mediates the NFκB pathway, and liver X receptor (LXR)-mediated JAK, liposome-mediated adenosine monophosphate-activated protein kinase (AMPK), and methylprednisolone sodium succinate (MPSS)-mediated TLR4 inhibit M1 microglia. While lithium salt mediates glycogen synthase kinase-3beta (GSK-3β) expression, simvastatin and monascin mediate peroxisome proliferator-activated receptor gamma (PPARγ) levels, fimasartan mediates caspase-1 levels, and minocycline upregulates matrix metalloproteinase 12 (MMP-12) expression.

Intracranial hematoma is a crucial factor contributing to brain injury after ICH. Mechanical damage is induced in adjacent tissues due to compression and dissection. Simultaneously, iron, heme, and cytotoxic hemoglobin can be passively released due to the lysis of erythrocytes adjacent to the hematoma (18). Microglial phagocytosis of hematoma occurs before erythrocytes lysis to protect the brain (19, 20). This process can be regulated by alternative activation of microglia *via* activating the CCR4/ERK/Nrf2 pathway and peroxisome proliferator-activated receptor γ (PPAR-γ) (21–23) (**Figure 3**).

There is ample evidence that microglial activation is essential for secondary damage to the brain after ICH (24). For example, microglia also produce pro-inflammatory factors (TNF-α, IL-1β, IL-6) and chemokines (CXCL2), which promote neuroinflammation (25, 26). Besides, the absorption of hematoma may also trigger a series of inflammatory reactions leading to primary and secondary brain damage (27).

## Microglial Polarization Following Ich

Although the terminology of “microglia polarization” is still widely used in the literature, most commonly in the M1 and M2 phenotypes. Complex high-content experiment and multi-omics technologies, including transcriptomic, epigenomic, and proteomics, have found novel microglia polarization states beyond the standard M1/M2 dichotomy, which leads to a fierce debate in microglial M1/M2 polarization in recent years (28).

As early as 2013, Chiu and colleagues utilized flow cytometry and deep RNA sequencing of acutely isolated spinal cord microglia. The study aimed to prove that microglial reactions must be interpreted in light of the tissue in which the activating stimulus is present (29). However, the M1/M2 model is not a pure phenomenon *in vivo*, as previously proven by Butovsky, by profiling CNS cells with an MG400 microglial chip (30), which ignores the crucial concept demonstrated by Chiu et al. (30). Besides, transcriptionally distinguishable subpopulations of microglia that appear to be a transcriptional continuum of the local population of microglia can be detected during homeostasis (31, 32), representing a transcriptional basis for the microglia phenotype diversity (33).

Single-cell RNA-sequence analysis of microglia suggested converged expression of M1 and M2 markers due to the influence of disease-related inflammatory processes (34). Furthermore, precise categorization of different microglia or monocyte subtypes based on specific types and stages of pathology or their relation to specific tissue injury types is also possible (35). In conclusion, a precise definition of microglial polarization has proven elusive, and the description of M1 or M2 phenotypes is an oversimplification of the complex biology of microglia.

In a recent transcriptional single-cell study, Keren-Shaul et al. found that microglial phenotypes other than the M1/M2 phenotypes exist. For example, a new subpopulation named disease-associated microglia (DAM) has been discovered through genome-wide transcriptomic analyses of microglia under different disease conditions. Although the gene profile of DAM and M1 microglia partially overlapped, the molecular signatures have shown apparent differences (36). Interestingly, DAM also exhibits anti-inflammatory/phagocytic and pro-inflammatory profiles (37). Activation of DAM depends on triggering receptors expressed on myeloid cells 2 (TREM2), a receptor located mainly on the surface of microglia. TREM2 promotes the phagocytosis of apoptotic neurons producing tiny quantities of pro-inflammatory cytokines (38). Research has also shown that TREM2 is activated in perihematomal areas, which improved attenuated neuroinflammation and neuronal apoptosis after ICH (39). Besides, according to a study done by Gao et al. in 2019, CEBPα, IRF1, and LXRβ are likely regulators of pro-inflammatory and anti-inflammatory DAM states. Based on emerging findings, it is possible to conclude that DAM represents a switch that substantially alters microglial function (40). While the DAM concept has been widely used in neurodegenerative diseases such as Alzheimer’s disease rather than ICH, it is clear that microglia is in a constant flux state and exquisitely sensitive to their environment.

Many studies have investigated the spatially and temporally restricted subsets of microglia during development and disease, further identifying the distinct molecular hallmarks and diverse cellular kinetics using massively parallel single-cell analysis and computational modeling (32, 41). For example, using single-cell RNA sequencing from human cerebral cortex samples, Olah et al. confirm the presence of four microglial subsets and elucidate the significance of subsets, such as the association with Alzheimer’s disease (AD) (42). More recently, Ochocka et al. demonstrate cellular and functional heterogeneity of microglia using flow cytometry and scRNAseq. In this experiment, multiple microglial clusters were obtained, and gene expression profiles underlying a specific cluster could reflect different functions. Hom-MG and activated microglia (Act-MG) were identified, which shows the distinct spatial distribution in experimental gliomas (43). Furthermore, an environment-dependent transcriptional network specifying microglia-specific programs have been developed, which identified substantial subsets of microglia associate with neurodegenerative and behavioral diseases (44).

In summary, microglia are activated by various pathologic events or changes in brain homeostasis, which are highly diverse and depend on the context and type of stressor or pathology. The complicated functional roles of microglia support the existence of distinct pro-inflammatory and anti-inflammatory functional states following ICH. The significance of defining microglial subtypes is to identify novel microglial functional conditions; determine the impact of molecules on microglia types, and discover ways to mediate functions in healthy physiology or disease (45). During the ICH progress, most investigators continue to use the expression of M1/M2 markers and microglia polarization as a surrogate for a genuine mechanistic understanding of how microglial function changes (46). Therefore, it would be interesting to identify the regulators and influencing factors that contribute to the polarization of microglia towards a neuroprotective or neurodestructive phenotype, which may shed new light on the pathogenetic role of microglia following ICH.

### Endogenous Mechanisms of Microglia

Much of the literature has emphasized the autoregulation of microglia during ICH progress. For example, studies by Wu et al. showed that soluble epoxide hydrolase expression is upregulated in microglia after ICH, which causes neuroinflammatory responses by degrading anti-inflammatory epoxyeicosatrienoic acid (47). Other studies have provided further evidence that microglial recruitment is associated with TWIK-related K+ channel 1 (TREK-1), which also triggers the secretion of pro-inflammatory factors such as IL-1β and TNF-α as well as cell adhesion molecules following ICH (48). Besides, low-density LRP1 in the neurovascular unit interacts with Mac-1 expressed by microglia to promote tPA-mediated activation of platelet-derived growth factor-cc (PDGF-cc). Activation of potential PDGF-cc and PDGF receptor-α signals can increase the permeability of the blood-brain barrier and deterioration following ICH (13).

Microglia have similarly been shown to be involved in anti-inflammatory and phagocytic effects on the hematoma, contributing to neurologic recovery after ICH. The correlation between regulatory T lymphocytes (Tregs) and neuroinflammatory response after ICH has been defined. In vitro experiments have demonstrated that Tregs modulate microglia polarization toward the anti-inflammation phenotype through the IL-10/GSK3β/PTEN axis in this regulatory process (49, 50).

#### The Regulatory Effect of miRNAs

As gene expression is regulated through genetic and epigenetic regulatory networks, there is growing evidence that miRNAs play essential roles in the microglial effects after ICH (51). For example, miRNA-7 (miR-7) can inhibit the expression of Toll-like receptor 4 (TLR4) and provoke a secondary microglia-mediated inflammatory response after ICH (52). Further studies have confirmed that agents that target TLR4 and miR-7, such as ligustilide (LIG) and senkyunolide H (SH), can exert neuroprotective effects against ICH by inhibiting Prx1/TLR4/NF-κB signaling *via* activation of microglia and astrocytes (53).

Recent studies have found that inhibition of miRNA-222 suppresses microglia-mediated inflammatory responses and improves neurological functions in a preclinical mouse model of ICH. Integrin subunit β8 (ITGB8) was identified as a directly negatively regulated target of miR−222 in microglial cells, leading to the attenuation of inflammation and apoptosis (54). Besides, miR-132 enhances the cholinergic blockade of the inflammatory response by targeting acetylcholinesterase (AChE), which also inhibits the activation of pro-inflammatory microglia and provides protection against neuronal death caused by ischemia (55).

Furthermore, as the critical factors in autophagy, miRNAs negatively regulate gene expression and autophagic activity of microglia. For example, miRNA-144 targets mTOR by directly interacting with 3′ untranslated regions (UTRs), which are involved in hemoglobin-mediated activation of microglial autophagy and inflammatory responses (56). The specific function of autophagy is dualistic and has been difficult to assess whether it has harmful or beneficial effects following ICH thus far. Despite many studies demonstrated that autophagy could enhance the protection of endoplasmic reticulum stress and reducing oxidative damage after ICH *via* clearing up the cell rubbish and oxidative-stress products (57, 58), recent studies showed autophagy positively regulates inflammation following ICH (59, 60).

#### Regulation of Microglia Function by Intracellular Signaling Following ICH

Anti-inflammatory microglia functions are accomplished by combining various signaling pathways that compose a complex network involved in multiple biological processes. Exploring the network of biological signaling pathways and its molecular basis contributes to novel interventions targeting signaling pathways that block the pathological progression of ICH (**Figure 3**).

#### The Roles of the AMPK Pathway and AdipoR1 in Microglia Function Following ICH

It has been demonstrated that adenosine monophosphate-activated protein kinase (AMPK) can drive the phenotypic shift from a pro-inflammatory state to an anti-inflammatory state (61). The expression of endogenous C1q/TNF-related protein 9 (CTRP9), an upstream trigger of the AMPK signaling pathway and an agonist of AdipoR1, is increased after ICH in animal models of long-term neurobehavior, peaking at 24 h after ICH. Further experiments have confirmed that the expression of AdipoR1 and p-AMPK can reduce the expression of inflammatory cytokines after ICH (62). Besides, the activation of MC4R also alleviates neurological deficits through the AMPK pathway following ICH, and interventions targeting MC4R, such as RO27-3225 administration, have been proven to be effective in animal experiments (63).

#### The Roles of the JNK Pathway in Microglia Function Following ICH

As mentioned above, Treg cells inhibit microglia-mediated inflammatory responses and improve neurological function *in vivo*, mainly by activate NF-κB through the JNK pathway (49, 64). There are controversies regarding the role of the JNK signaling pathway following ICH, which has received increased attention in the clinic in recent years. For example, the synthesis of the liver X receptor (LXR) agonist TO901317 was shown to exert specific effects in an ICH model by inhibiting JNK signaling. The hyperbaric oxygen preconditioning (HBOP) model of ICH has demonstrated the potential relevance between JNK phosphorylation and the immunological activity of anti-inflammatory microglia (65). As current practical limitations include drug side effects, uncertainties regarding efficacy, surgical injuries, and complications, there are no standardized clinical interventions for ICH except intracranial pressure-lowering therapies. Therefore, hyperbaric oxygen therapy provides a feasible alternative intervention with mild adverse effects against ICH, and the mechanism of HBOP in ICH needs further exploration and verification.

#### The Impact of the Toll-like Receptor 4 (TLR-4) Pathway on Microglia Function

Toll-like receptor 4 (TLR4) plays a crucial role in the innate immune response. It can be concluded that loss of TLR4 reduces the recruitment of pro-inflammatory microglia and markedly alleviates inflammation around the hematoma in the animal model (66). Further studies have shown that TLR4 also inhibits the phagocytosis of microglia on the surface of red blood cells, resulting in hematoma absorption delay and severe neurological deficits in ICH patients (67). TLR4-mediated autophagy of microglial activation contributes to secondary brain injury and brain recovery and inflammatory damage following ICH (68).

The role of TLR4 in secondary brain damage following ICH has been elaborated in detail, and therapeutic strategies targeting TLR4 are relatively well-developed. Therefore, TLR4 remains a promising target for inhibiting undesired microglial responses, and interventions targeting TLR4-related pathways may represent future candidates for ICH therapy.

### Regulation of Microglia Function by Extracellular Signals Following ICH

As the regulatory effects of intracellular signaling pathways on microglia after ICH have been discussed, the final section of this paper addresses how extracellular signaling regulators influence microglial morphology and function. Compared with intracellular signals, extracellular signals are more complicated and vulnerable to disruption, which means they have the potential to be translated into clinically effective targeted therapies for ICH.

#### Interventions Targeting Microglia Functions for ICH

##### Interleukins

The interleukins (ILs) level is intimately associated with the development and progression of ICH, which may be achieved by modulation of microglia functions. For example, activation of the IL-4/transcription 6 (STAT6) axis improved long-term functional recovery in a mouse model of ICH (69). Conversely, expression of IL-15 exacerbates brain injury following ICH by mediate the crosstalk between microglia and astrocytes (70).

Besides, antibodies against IL-17A can prevent ICH-induced expression of TNF-α, IL-1β, and IL-6 and inhibit microglial activation (71). Further examination revealed that IL-17A promotes autophagy in pro-inflammatory microglia, thus maintaining the body’s normal immune response and alleviating brain edema after ICH (60). Notably, recent studies have found that intraventricular infusion of IL-33 can alleviate neurological deficits following ICH by promoting the transformation of pro-inflammatory microglia (72). Deferroxamine (DFA) can also inhibit the activation of pro-inflammatory microglia by downregulating IL-1β and TNF expression, reducing secondary brain insult following ICH.

#### Nuclear Factor-κB

Evidence has suggested that NF-κB translocates to the nucleus, and pro-inflammatory mediators (NO, TNF-a, and IL-6) are produced following inflammatory response after ICH. These results suggest that combined targeting of NF-κB signaling pathway inhibition may be a more effective anti-neuroinflammatory strategy following ICH (53).

Regulation of NF-κB activity may also have promising clinical benefits following ICH. Analysis of thrombin toxicity *in vitro* shows that has thrombin release after ICH led to the increased expression of NF-κB in microglia (73, 74). Treatment modalities disrupting this harmful process, such as miR-181c mimic therapy, are expected to regulate thrombin-driven inflammation after cerebral hemorrhage (75).

#### Glycogen Synthase Kinase-3β

It is widely acknowledged that glycogen synthase kinase-3beta (GSK-3β) exerts a potent pro-inflammatory effect following ICH (76). Studies have shown that the hematoma volume is significantly decreased by GSK-3β inhibition after ICH due to enhanced microglia-mediated phagocytosis (77). Consistently, the GSK-3β inhibitor 6-bromoindirubin-3′-oxime (BIO) has been shown to relieve inflammation by blocking GSK-3β Tyr216 phosphorylation/activation following ICH. BIO may exert a protective effect against ICH by increasing the number of anti-inflammatory microglia through inactivating GSK-3β (78).

It is interesting to note that the molecular mechanism by which lithium salt can treat ICH in clinical practice has already been elucidated. Recently, it has been shown that LiCl treatment decreased the death of mature oligodendrocytes (OLGs) in ICH mice, which may be regulated by the LiCl-induced inhibition of glycogen synthase kinase-3β (GSK-3β) (79).

#### Peroxisome Proliferator-Activated Receptor Gamma (PPAR-γ)

The phagocytic activity of microglia is required to remove the hematoma after ICH; however, the pro-inflammatory mediators and free radicals released as a result of microglial activation and phagocytosis are toxic to neighboring cells and lead to secondary brain damage following ICH (80). ICH mouse model demonstrated that peroxisome proliferator-activated receptor gamma (PPAR-γ) prevents LPS‐induced pro-inflammatory microglial activation while facilitating microglial polarization towards the anti-inflammatory phenotype (81). Besides, PPAR-γ promotes phagocytosis in a timely and effective manner, limiting the toxic effects of hemolysis by facilitating hematoma clearance following ICH (82).

Studies have demonstrated that PPAR-γ activation is imperative for enhancing the phagocytic ability of anti-inflammatory microglia by CD36 (18). Furthermore, 15(S)-hydroxyeicosatetraenoic acid, an exogenous PPAR-γ agonist, improves functional recovery following ICH and exerts neuroprotective effects (83).

Based on in-depth basic research, PPAR-γ agonists have been widely used in clinical treatment. For example, the neuroprotective effects of statins following ICH through PPAR-γ activation and enhancement of microglia-induced erythrocyte phagocytosis have been established (84). Besides, monascin, as a novel dual agonist of PPAR-γ and Nrf2, facilitates microglial phagocytosis of the hematoma and exerts neuroprotective effects following ICH (85, 86).

#### Caspase Family

Caspase-mediated cascades play an essential role in mediating anti-inflammatory microglial death (87). For example, AC-YVAD-CMK can alleviate brain edema by inhibiting the activation of pro-caspase-1 and downregulating the expression of inflammation-related factors, which is accompanied by decreasing activated microglia at 24 h post-ICH (88).

Clinical studies have found that fimasartan (an angiotensin II receptor blocker) significantly reduces the activation of the caspase-1 pathway after ICH (89), suggesting that it is effective in ICH by regulating caspase-1–mediated microglial autophagy.

#### Matrix Metalloproteinases (MMPs)

The first serious discussions of MMP-12, which is harmful and contributes to secondary damage after ICH, emerged in 2005 (90). Subsequent studies found that MMP-9 binds to injured neurons in culture, activates pro-inflammatory microglia, and exerts neurotoxic effects after ICH (91). Based on this, further research proposed that inhibition of MMP-9 improves prognosis following ICH (92).

MMP-mediated microglial activation has become a potential therapeutic target for ICH. For example, minocycline, a widely available drug that alleviates brain damage, effectively reduces early upregulation of MMP-12 expression (93, 94)and induces anti-inflammation microglial polarization, which reduces the levels of inflammatory cytokines and the number of microglia surrounding the hematoma after ICH (86). It should be noted that although the molecular mechanism is unclear, MMP-12 expression and microglial infiltration around the hematoma are significantly reduced after stem cell transplantation following ICH (95, 96).

#### Iron Chelators

Iron overload is a significant cause of brain damage because iron toxicity contributes to pro-inflammatory microglial activation following collagenase-induced ICH. Therefore, reducing the accumulation of iron can moderately improve the outcomes after ICH (97). As an iron chelator, minocycline can reduce free iron and iron handling protein levels, thus prevent neuronal death (98). Besides, VK-28, a brain-permeable iron chelator, is superior to and less toxic than DFA following ICH (99, 100).

**Figure 4:**
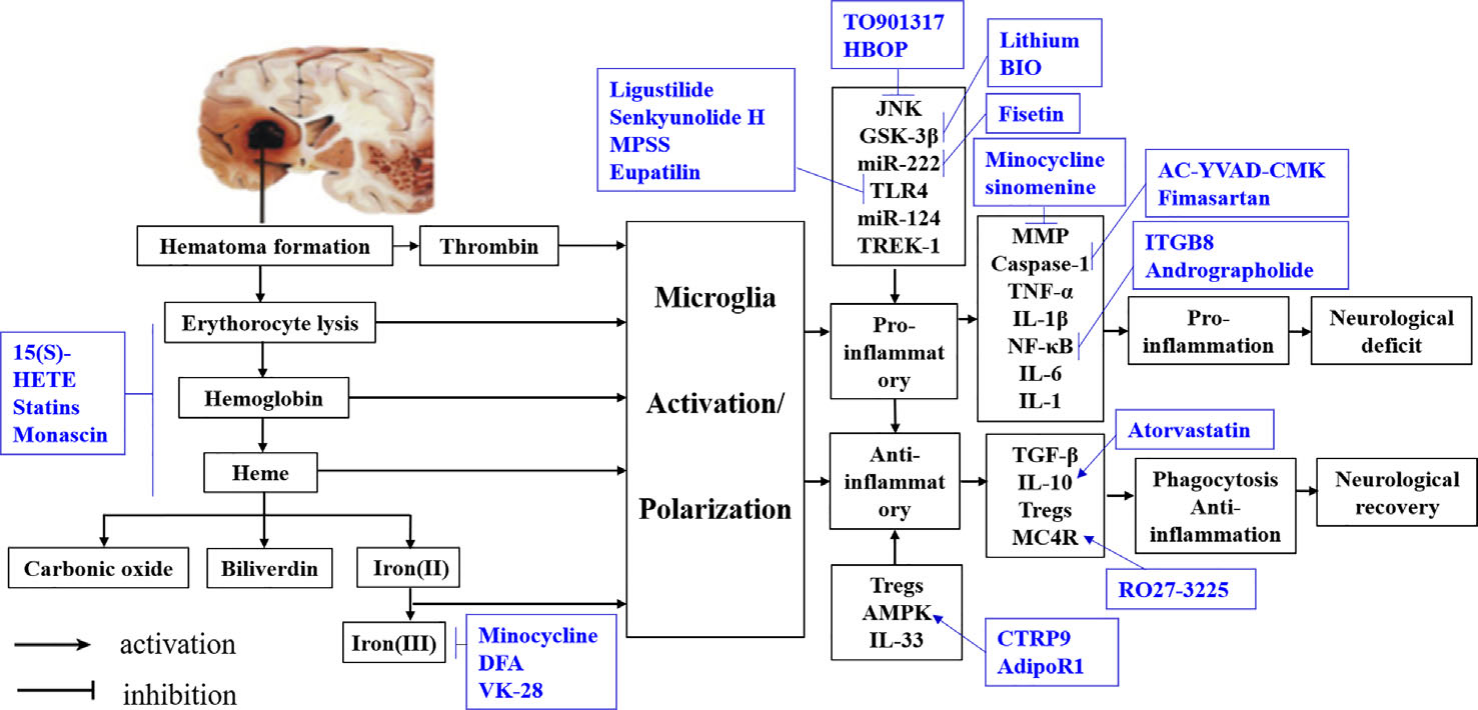
Summary of interventions for ICH targeting microglial polarization. Available intervention strategies are labeled blue.

The evidence from observational studies shows that microglia function is controlled by complex regulatory networks (**Table 1**), an understanding of which is critical for elucidating phenotypic and genotypic variations in microglia and developing therapeutic interventions for ICH (**Figure 4**).

**Table 1:** Potential interventions for microglia polarization after intracerebral hemorrhage.

Phenotype	Activating signals (events)	Markers (events)	Result	Purpose
Pro-inflammatory microglia	JNK (TO901317, HBOP -)GSK-3β (Lithium, BIO -) miR-222 (Fisetin -) TLR4 (Ligustilide, Senkyunolide H, MPSS, Eupatilin -)miR-124 TREK-1	MMP (Minocycline, sinomenine -) Caspase-1 (AC-YVAD-CMK, Fimasartan -) TNF-αIL-1βNF-κB (ITGB8, Andrographolide -) IL-6 IL-1	Pro-inflammation	Neurological deficit
Anti-inflammatory microglia	TregsAMPK (CTRP9, AdipoR1 +)IL-33	TGF-βIL-10 (Atorvastatin +)Tregs MC4R (RO27-3225 +)	Phagocytosis Anti-inflammation	Neurological recovery

## Potential Therapeutic Strategies Targeting Microglia Function After Ich

Exploring the regulatory mechanism of microglial immunophenotype changes may help identify the hematoma scavenging mechanism and a precise therapeutic target for ICH. The multi-omics technologies have made significant achievements in the research of microglial activation (101). The application of systematic multi-omics approaches to precision medicine and systems biology has great potential to improve the care of patients with ICH. Notably, the target gene identified by multi-omics studies can potentially be used for drug repositioning in ICH, which is approved to be cheaper, quicker, and effective (102).

## Conclusion

This review aimed to objectively discuss and assess the role of microglia in regulating neuronal injury after ICH. The findings indicate that pro-inflammatory or anti-inflammatory microglia have divergent effects, which have significant implications for understanding microglia function *via* intracellular and extracellular signal-regulated pathways. Besides, this review provides the first comprehensive assessment of cellular and molecular mechanisms and pathways responsible for regulating microglia, including an in-depth analysis of signaling pathways strongly associated with microglia following ICH. Notwithstanding the relatively limited number of reliable clinical trials and the lack of molecular genetic studies on the phenotypic change of microglia, this work offers valuable insights into a novel therapeutic strategy for ICH that targets microglia. Further research on interventions associated with microglial physiology is an essential next step in confirming a framework for assessing the feasibility of the novel therapy mentioned above.

## Data Availability Statement

The data that support the findings of this study are openly available in pubmed at https://pubmed.ncbi.nlm.nih.gov/. Data sharing is not applicable to this article as no new data were created or analyzed in this study.

## Author Contributions

All authors listed have made a substantial, direct and intellectual contribution to the work, and approved it for publication. All authors contributed to the article and approved the submitted version.

## Funding

This work was supported by a grant from National Natural Science Foundation of China (81771294) and the National undergraduate innovation training project (2020105330289).

## Conflict of Interest

The authors declare that the research was conducted in the absence of any commercial or financial relationships that could be construed as a potential conflict of interest.
